# Identification and Validation of miRNA-TF-mRNA Regulatory Networks in Uterine Fibroids

**DOI:** 10.3389/fbioe.2022.856745

**Published:** 2022-03-22

**Authors:** Xiaotong Peng, Yanqun Mo, Junliang Liu, Huining Liu, Shuo Wang

**Affiliations:** ^1^ Department of Gynaecology and Obstetrics, Xiangya Hospital, Central South University, Changsha, China; ^2^ Department of Orthopaedics, Shanghai Jiaotong University Affiliated Sixth People’s Hospital, Shanghai, China

**Keywords:** uterine fibroids, miRNA-TF-mRNA, regulatory network, bioinformatics, biomarkers

## Abstract

Uterine fibroids (UF) are the most common benign gynecologic tumors and lead to heavy menstrual bleeding, severe anemia, abdominal pain, and infertility, which seriously harm a women’s health. Unfortunately, the regulatory mechanisms of UF have not been elucidated. Recent studies have demonstrated that miRNAs play a vital role in the development of uterine fibroids. As a high-throughput technology, microarray is utilized to identify differentially expressed genes (DEGs) and miRNAs (DEMs) between UF and myometrium. We identified 373 candidate DEGs and the top 100 DEMs. Function enrichment analysis showed that candidate DEGs were mainly enriched in biological adhesion, locomotion and cell migration, and collagen-containing extracellular matrix. Subsequently, protein-protein interaction (PPI) networks are constructed to analyze the functional interaction between DEGs and screen hub DEGs. Subsequently, the expression levels of hub DEGs were validated by real-time PCR of clinical UF samples. The DGIdb database was used to select candidate drugs for hub DEGs. Molecular docking was applied to test the affinity between proteins and drugs. Furthermore, target genes for 100 candidate DEMs were predicted by miRwalk3.0. After overlapping with 373 candidate DEGs, 28 differentially expressed target genes (DEGTs) were obtained. A miRNA-mRNA network was constructed to investigate the interactions between miRNA and mRNA. Additionally, two miRNAs (hsa-miR-381-3p and hsa-miR-181b-5p) were identified as hub DEMs and validated through RT-PCR. In order to better elucidate the pathogenesis of UF and the synergistic effect between miRNA and transcription factor (TF), we constructed a miRNA-TF-mRNA regulatory network. Meanwhile, *in vitro* results suggested that dysregulated hub DEMs were associated with the proliferation, migration, and apoptosis of UF cells. Our findings provided a novel horizon to reveal the internal mechanism and novel targets for the diagnosis and treatment of UF.

## Introduction

Uterine fibroids (UF) are recognized as the most common benign tumors of the female internal genitalia (Pitter et al., 2013). The cumulative incidence of UF in women before the age of 50 is as high as 70% (Walker and Stewart, 2005). Despite their benign nature, UF still causes a variety of symptoms, such as pelvic pressure, uterine bleeding, anemia, or infertility (Islam et al., 2013), and is mainly treated through surgery and drugs (Zhang L. et al., 2021), such as hysterectomy, leading to devastating effects on approximately two 200,000 per year in United States (Cardozo et al., 2012). Unfortunately, their origins and internal mechanism have not been characterized so far (Andersen and Barbieri, 1995). Therefore, it is urgent to reveal the pathogenesis of UF and identify novel biomarkers for early screening, diagnosis, and individualized precision therapy.

At the post-transcriptional level, microRNAs (miRNAs) are single-stranded RNA molecules (approximately 18–25 nucleotides long) and negatively regulate gene expression at the post-transcriptional level through binding to complementary sites in the 3ʹ-untranslated regions (3′UTR) of target genes, resulting in messenger RNAs (mRNAs) translational arrest or degradation (Rupaimoole and Slack, 2017). The above processes are similar to the regulation mechanism of transcription factors (TFs) through binding to a TF binding site in the promoter region, which suggests that they share a mutual binding mechanism. Recently, there has been increasing evidence that miRNA can regulate more than 30% of the encoding genes in mammals and play a vital role in the pathogenesis and development of various human diseases, including UF (Ali et al., 2020). For example, downregulated miR-139-5p contributed to the progression of UF through collagen Ⅰ deposition and p38 phosphorylation (Ahn et al., 2021). The caspase-3 cleavage and elongation factor-2 phosphorylation were activated by miR-21, leading to UF cell apoptosis and stalled translation (Fitzgerald et al., 2012). Furthermore, transfected leiomyoma cells with miR-150–5p mimics had stronger migrational ability and secreted more collagen through p27^Kip1^ activation and Akt inhibition (Lee et al., 2019). Hence, miRNA–mRNA regulatory networks are strongly associated with tumorigenesis and progression of UF. However, the specific microRNAs in UF and their potential role on target genes or transcription factors (TFs) regulatory networks have remained unrevealed.

**SCHEME 1:**
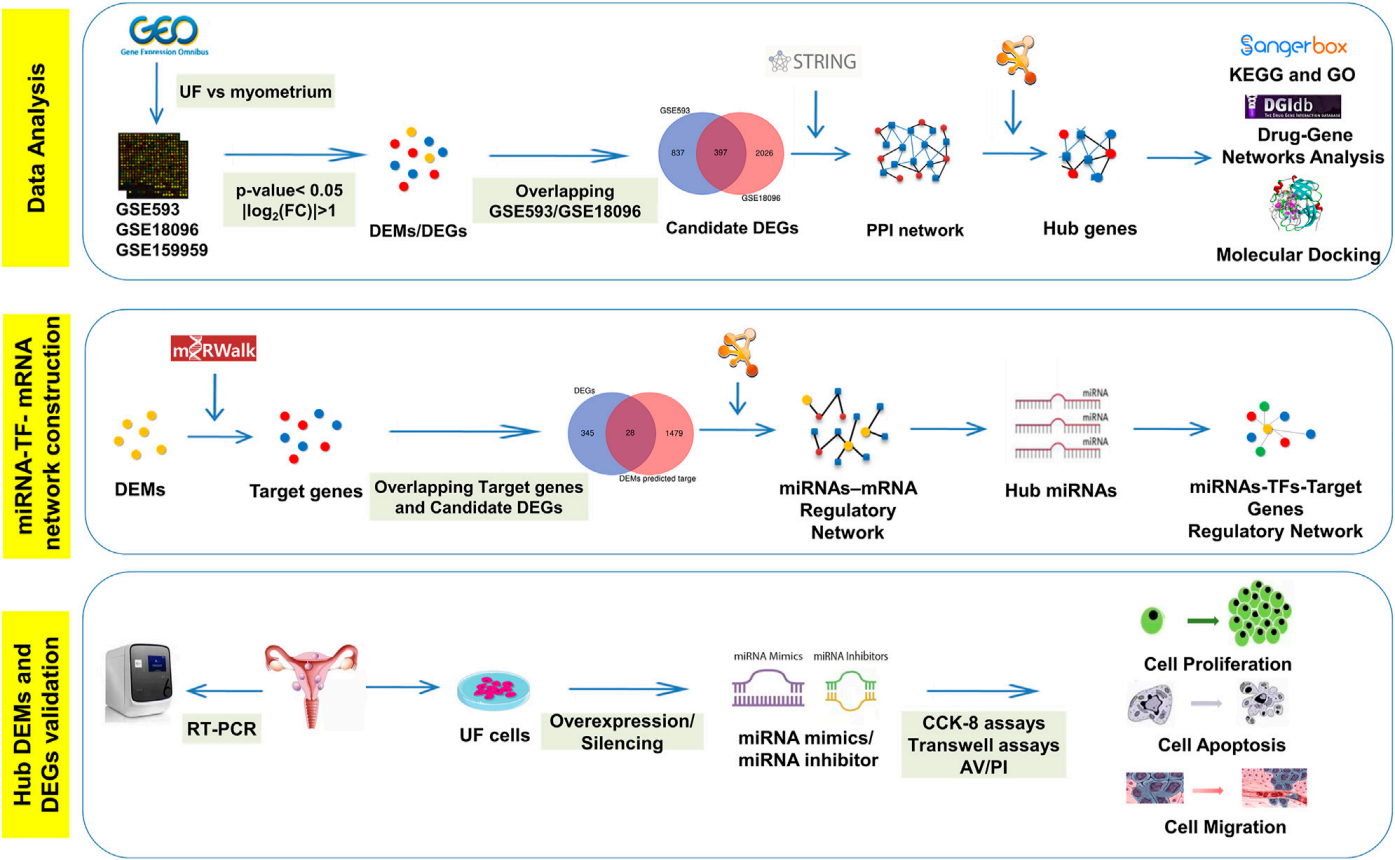
The schematic workflow of current study.

Benefiting from bioinformatics and high-throughput sequencing technology’s rapid development, network-based methods emerge and expand our knowledge of regulatory relationships among genes (Fu and Dong, 2018). Regulatory networks have been widely applied to screen pathogenic genes, elucidate miRNAs’ functions, and provide targeted drugs for disease treatment for subsequent experimental verification (Hu et al., 2017). As shown in Scheme 1, differentially expressed genes (DEGs) and miRNAs (DEMs) in UF tissues versus normal myometrium tissues were identified through analyzing two mRNA microarrays (GSE593 and GSE18096) and one miRNA microarray (GSE159959) downloaded from gene expression omnibus (GEO) databases. Subsequently, we performed functional annotation, enrichment analysis, and established protein-protein interactions (PPI) and drug-hub DEGs networks. Real-time PCR (RT-PCR) and molecular docking were used to check the DEG expression based on human tissue samples for diagnosis and screen effective target-drugs respectively. Furthermore, 28 differentially expressed target genes (DETGs) out of the top 100 DEMs were obtained from the intersection between predicted target genes and DEGs. MiRNA-target gene regulatory networks associated with UF were established and provided the interactions that have not yet been reported. Hub DEMs’ functional annotation and pathway enrichment analysis were also implemented. We identified the interaction between TFs and hub DEMs to reveal a common regulatory mechanism. Combining with the above bioinformatics results, we designed mimics and inhibitors of hub DEMs and evaluated their functions on the proliferation, migration, and apoptosis of UF cells. Taken together, our study looked forward to providing a novel horizon for UF treatment through unscrambling the miRNA-TF-mRNA regulatory network, resulting in the avoidance of irretrievable harm to female reproductive health.

## Materials and Methods

### Data Extraction

**FIGURE 1:**
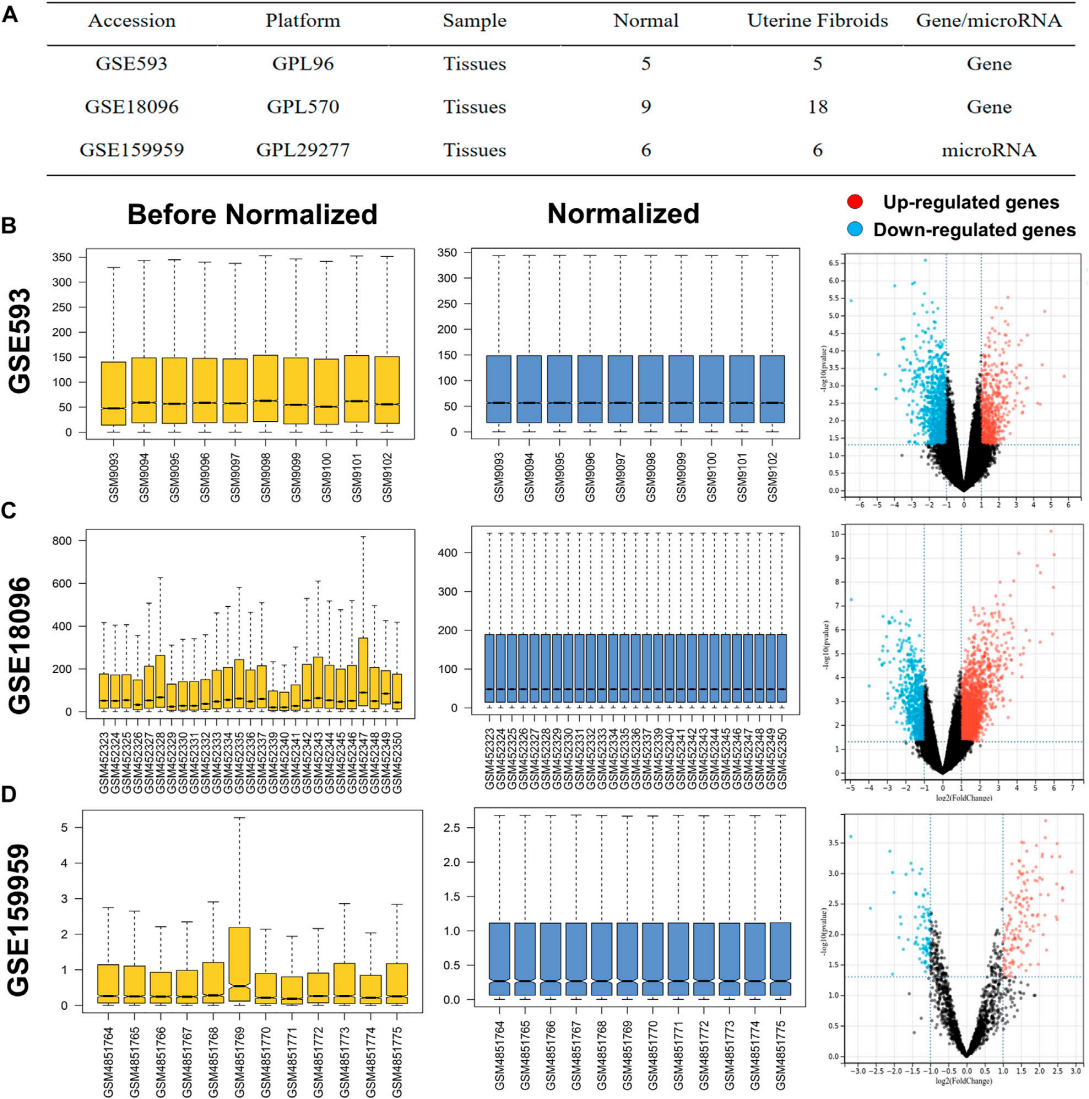
**(A)** Characteristics of mRNA and miRNA expression profiles of UF. The before and after normalization and volcano plots of **(B)** GSE593, **(C)** GSE159959 and **(D)** GSE18096.

UF-related expression profiles for miRNA (GSE159959) and mRNA (GSE593) and GSE18096) were collected from the gene expression omnibus (GEO) database (https://www.ncbi.nlm.nih.gov/geo/). The associated information from the above three datasets was shown in Figure 1A. Due to the different process platforms of the included datasets, we normalized these data using R’s limma package.

### Analysis of DEMs and DEGs

The normalized expression datasets were analyzed using the GEO2R (http://www.ncbi.nlm.nih.gov/geo/geo2r/) for comparison between UF tissues and matched myometrium tissues. The screening criteria were set as *p*-value <0.05 and |log2-fold change (FC)| > 1, the top 100 regulated miRNAs were selected as DEMs. With the same threshold, the upregulated and downregulated overlapping DEGs were obtained between GSE593 and GSE18096. The DEMs and DEGs in the respective profiles were visualized in volcano plots. Furthermore, heatmaps of DEMs and DEGs were visualized by TB tools, which is a toolkit that integrates various biological data-handling tools.

### Construction of the PPI Network and Analysis of Modules

The online STRING database (https://string-db.org) was used to visualize the positive functional interaction between DEGs and the threshold (combined scores ≥0.4). Cytoscape software vision 3.8.2 was utilized to screen the upregulated and downregulated DEGs and construct the PPI network. Additionally, molecular complex detection (MCODE) v1.5.1.16 (Bader and Hogue, 2003) was used to identify hub genes with the following cut-off criteria: degree cut-off = 2, max. depth = 100, node score cut-off = 0.2, and k-Core = 2.

### Functional Enrichment Analysis

To further clarify the functional annotation of DEGs and hub genes, Gene Ontology (GO) functional annotation and Kyoto Encyclopedia of Genes and Genomes (KEGG) pathway enrichment analysis were conducted in the Sangerbox database (http://sangerbox.com/Tool). GO functional analysis was mainly classified into cellular components (CC), biological processes (BP), and molecular functions (MF).

### Drug-Hub Gene Network Analysis

In order to explore the target drugs of hub genes, the drug-gene interaction database (DGIdb, https://dgidb.genome.wustl.edu/) was used, which is the online tool for drug-gene predication. Finally, Cytoscape was used to construct a drug-gene network.

### Homology Modeling and Molecular Docking

Recently, artificial intelligence brought revolutionary changes to the field of protein structural biology through convolutional neural networks. As an AI prediction tool, Alphafold (https://alphafold.ebi.ac.uk/), as an AI prediction tool, predicts highly accurate protein structures competitive with experiments based on their amino acid sequence. Therefore, despite the crystal structures of human BIRC5 and TYMS having yet to be solved in the RCSB protein data bank (PDB), we adopted Alphafold to build the structure of STAT6 through in silico modelling. From the amino acid sequence of human BIRC5 and TYMS (accession numbers: O15392 and P04818, respectively) from the UniProt-KB database (http://www.uniprot.org/) and predicted protein structures starting from the sequence through AlphaFold v2.0. The stereochemical quality of the predicted model was evaluated by the local distance difference test (LDDT) score in the AlphaFold database.

Molecular docking simulation is a technique to predict the first-rank orientation of small-molecules to macromolecule targets to form stabilized complexes. Associated small-molecules were both downloaded from the Pubchem database (https://pubchem.ncbi.nlm.nih.gov). The AutoDockTools and PyMol were applied to preprocess the input file, including hydrogenation and deletion of crystallographic water and ligands. Molecular docking between small-molecules and BIRC5, TGFBR2, and TYMS binding pockets was performed by Autodock Vina with default parameters. The predicted binding interaction geometries of irinotecan (aka irinotecan hydrochloride) and BIRC5 or TYMS were visualized and the docking affinity between small-molecules and protein targets was scored. The optimal docking conformation and related results were analyzed by PyMol.

### Prediction of Target Genes for DEMs and Construction of miRNA-Target Gene Regulatory Network

MiRWalk3.0 (http://zmf.umm.uni-heidelberg.de/apps/zmf/mirwalk3/) is an online tool to predict the downstream target genes of DEMs. Then, differentially expressed target genes (DETGs) were obtained by overlapping the predicted genes and DEGs for further analysis. In addition, the miRNA-target gene regulatory networks were visualized by Cytoscape. Among the DEMs in this network, we selected the DEMs with the highest number of regulatory genes ranking top two as the hub DEMs.

### Functional and Pathway Enrichment Analysis of Hub DEMs

To further analyze the function of hub DEMs, GO and KEGG pathways were performed using miRPath v3.0 (http://snf-515788.vm.okeanos.grnet.gr/). An adjusted *p* < 0.05 was considered significantly enriched.

### Establishment of the miRNA-TFs-Target Gene Networks

It has been confirmed that TF-mediated transcriptional regulation and miRNA-mediated post-transcriptional regulation are tightly coupled, which implies that they share a common regulatory mechanism (Lewis et al., 2005). Transcription factors and miRNAs are known to regulate each other as well as their target genes. Therefore, further research on the regulatory relationship between TFs and miRNAs can help elucidate the underlying mechanism of tumorigenesis and progress. Based on the hub DEMs, we predicted the potential TFs *via* the iRegulon plug-in (Lewis et al., 2005). Relevant parameters were as follows: enrichment score threshold = 5.0, ROC threshold for AUC calculation = 0.05, rank threshold = 5,000, minimum identity between orthologous genes = 0.05, and maximum FDR on motif similarity = 0.001. The Cytoscape software was subsequently utilized to visualize miRNA-TF-target gene regulatory networks.

### Tissue Samples

Ten cases of UF tissue and matched normal myometrial tissue samples were collected from patients who underwent myomectomy in Xiangya Hospital from January 2021 to January 2022, aged 36–49 years old. All patients had regular menstruation without other complications, and they did not take hormone drugs within 3 months before surgery. All samples were taken with the consent of the patients, and informed consent was signed. Consent was granted by all women included in the study, and informed consent was signed. Tissues were stored at −80°C immediately after harvest until further use. The pathological diagnosis of the tissue was confirmed by at least two independent pathologists.

### Hematoxylin-Eosin Staining

The tissues were fixed for 24 h and dehydrated with gradient alcohol. Paraffin-embedded tissues were prepared and subsequently cut into 5 µm slices, which were then stained using the H&E staining kit (Beijing Solarbio Science and Technology Co., Ltd., Beijing, China). The pathological changes in UF were observed and captured with five random images under a light microscope (Leica DMi6-M, Leica Microsystems Co., Ltd.).

### Isolation and Culture of Human Leiomyoma Cells and Myometrial Smooth Muscle Cells.

The human UF tissue was washed three times with PBS solution and minced into small pieces (3–6 mm^3^) with sterilized eye scissors. To obtain single-cell suspensions, the tissue fragments were incubated with Dulbecco’s modified eagle media (DMEM, Hyclone, United States) medium supplemented with 4 mg/ml type I collagenase (Sigma-Aldrich, United States) and 1 mg/ml DNase I (Sigma-Aldrich, United States) at 37°C in a humidified incubator with 5% CO2 for 2 h. Subsequently, the suspensions were passed through a 40-μm cell strainer (Corning, United States) to eliminate undigested tissue. The primary cells were cultivated in DMEM medium containing 10% fetal bovine serum (FBS, Gibco, United States), 100 U/ml penicillin, and 100 μg/ml streptomycin at 37°C in a 5% CO2 incubator. Cultured human primary cells were passaged every 4–6 days and analyzed at the third passage for further study.

### Cell Transfection

To explore the function of DEMs in leiomyoma cells, the primary cells were seeded in 6-well plates at an appropriate density. When cells were in log-phase growth, cell transfection was performed with negative control (NC) or hub DEM mimics or inhibitors (50 nM) using Lipofectamine 3000 (Invitrogen, United States). The sequences were synthesized by GenePharma (Shanghai, China).

### RNA Extraction and RT-PCR Validation

RNA was extracted from tissues using the TRIZOL method (TransGen Biotech, Beijing, China) and the RNA was reverse transcribed into cDNA according to the instructions of the NovoScript® II reverse transcriptase (Novoprotein, Shanghai, China). MiRNA was extracted using the miRNA extraction kit (Tiangen, Beijing, China) and reversely transcribed into cDNA through the polyA tailing method. RT-PCR was carried out by using SYBR® Green MasterMix (Takara, Japan) for hub DEGs and miRNA universal SYBR qPCR Master Mix (Vazyme Biotech, Nanjing, China) for hub DEMs in an ABI PRISM® 7500 Sequence Detection System (Applied Biosystems Inc: Foster City, CA). RT-PCR reaction system: For hub DEGs, 45 s at 95°C, 40 cycles for 30 s at 95°C, 45 s at 60°C, 60 s at 72°C and a final cycle for 10 min at 72°C; For hub DEMs, 5 min at 95°C, 40 cycles for 10 s at 95°C, 30 s at 60°C. Fluorescence values were collected. Replicate wells were set up for each sample. GAPDH and U6 were used as the internal reference for hub DEGs and DEMs detection, respectively, and the experiment was repeated three times independently. The mean CT value was calculated. Relative quantification (ΔΔCT method) was applied for semi-quantitative analysis, and the target gene expression was represented by 2^−ΔΔCT^ values. The sequences of the primer were synthesized by Sangon Biotech (Shanghai, China).

### CCK-8 Assay

Cells were transfected for 48 h and then inoculated into 96-well plates at a density of 4 × 10^3^/well. The cell proliferation was measured at 1–3 days, respectively. At each time point, 10 μl of CCK-8 solution (New Cell and Molecular Biotech, Jiangsu, China) was added to each well and incubated in the incubator for 2 h. Finally, the absorbance at 450 nm, as the optical density (OD) of the samples, was detected by spectrophotometry at different time points.

### Cell Migration Assay

After 24 h of transfection of mimic or inhibitor, we trypsinized and resuspended to obtain a single cell suspension. The upper layer of the Transwell chamber was added with FBS-free suspension at a cell density of 3 × 10^4^/well. The lower layer was added with 20% FBS medium to drive cell migration. After 24 h of culture, we used cotton swabs to remove the nonmigrated cells in the upper chamber, and migrated cells in the lower chamber were fixed and stained with 0.1% crystal violet. Five randomly selected microscopic views were recorded through a light microscope and analysed by ImageJ software to count the number of cell migrations.

### Apoptosis Detection

To determine the effect of hub DEMs on the apoptosis of leiomyoma cells, cells were harvested and centrifuged for annexin V-FITC/PI apoptosis detection (Boster Biotech Co., Ltd., Wuhan, China) after 24 h of transfection. After being washed twice, transfected cells were incubated with 5 µl of 488-annexin V and 5 µl of PI for 15 min at room temperature and protected from light. Subsequently, the flow cytometer (BD, United States) was applied to detect the apoptosis rates in each group. The apoptosis rate = sum of the early and late apoptotic rates.

### Statistical Analysis

Statistical analysis was carried out using SPSS 16.0 statistical software. Data was expressed as mean ± SD, and the differences between the two groups were compared using the independent sample *t*-test. The differences between the two groups were compared using the independent sample t-test, and the differences were considered statistically significant at *p* < 0.05.

## Results

### Identification of DEGs and DEMs in UF

**FIGURE 2:**
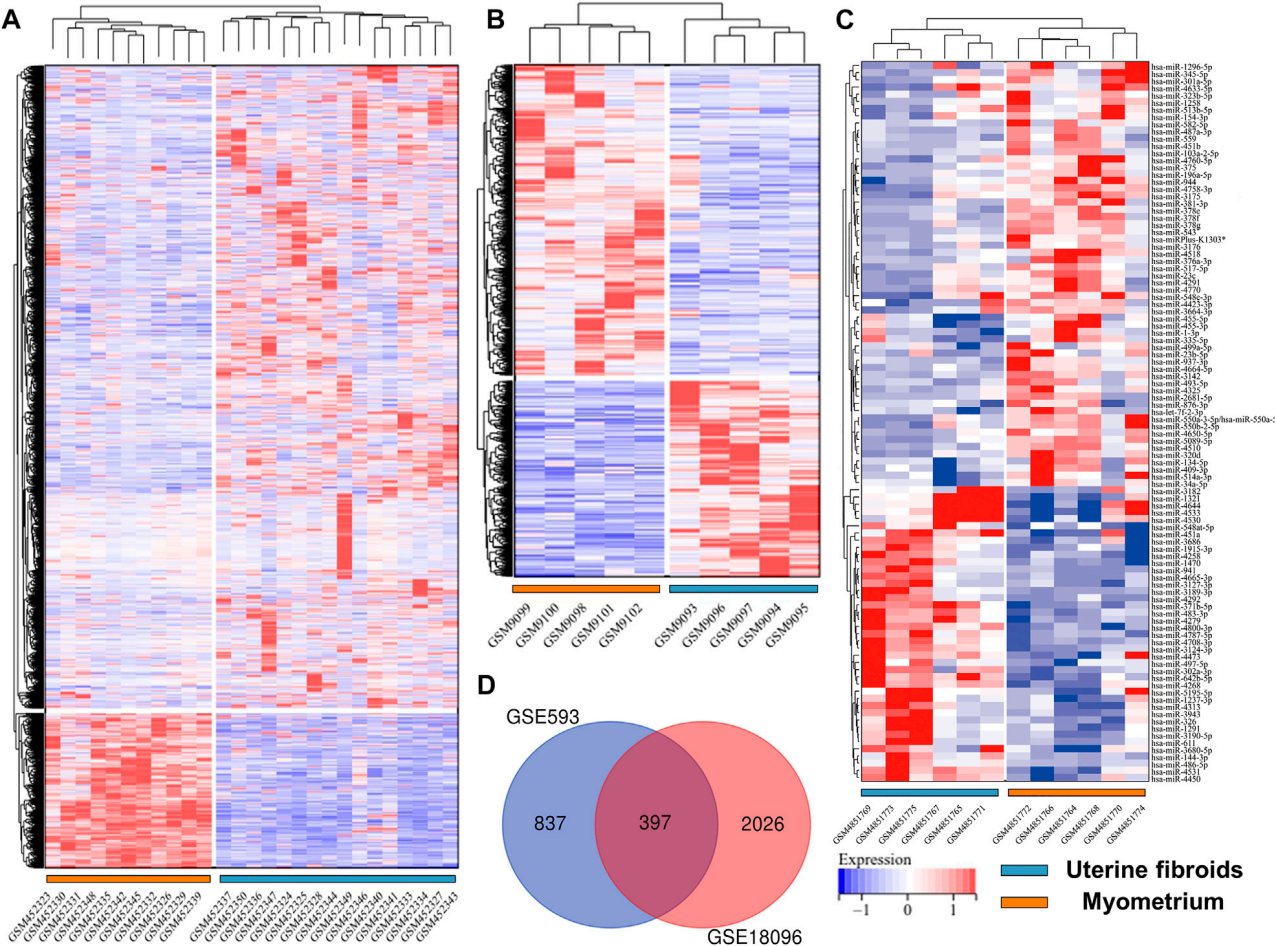
Heatmap of **(A)** GSE18096. **(B)** GSE593, **(C)** GSE159959. **(D)** Venn diagram of screening DEGs form mRNA expression profile.

Before screening DEGs and DEMs in UF, the three independent expression datasets processed on different platforms were normalized. Based on the mentioned threshold, differential expression analysis was applied to obtain DEGs and DEMs. 1185 and 2054 DEGs and 229 DEMs were identified in GSE593, GSE18096, and GSE159959, respectively. To shrink the scope of DEM screening, DEMs whose |log2 FC| ranked in the top 100 were chosen as candidate DEMs. As shown in Figures 1B–D, 2A–C, volcano plots and heatmaps exhibited the overall distribution of DEGs and DEMs for UF versus normal myometrium. Furthermore, a total of 397 candidate DEGs were identified by Venn diagram (Chen and Boutros, 2011) analysis through the intersection of these two mRNA datasets (Figure 2D). Excluding DEGs with inconsistent expression trends in these two datasets, 373 genes containing 192 upregulated and 181 downregulated genes were obtained as candidate DEGs.

### PPI Network Construction and Module Analysis

**FIGURE 3:**
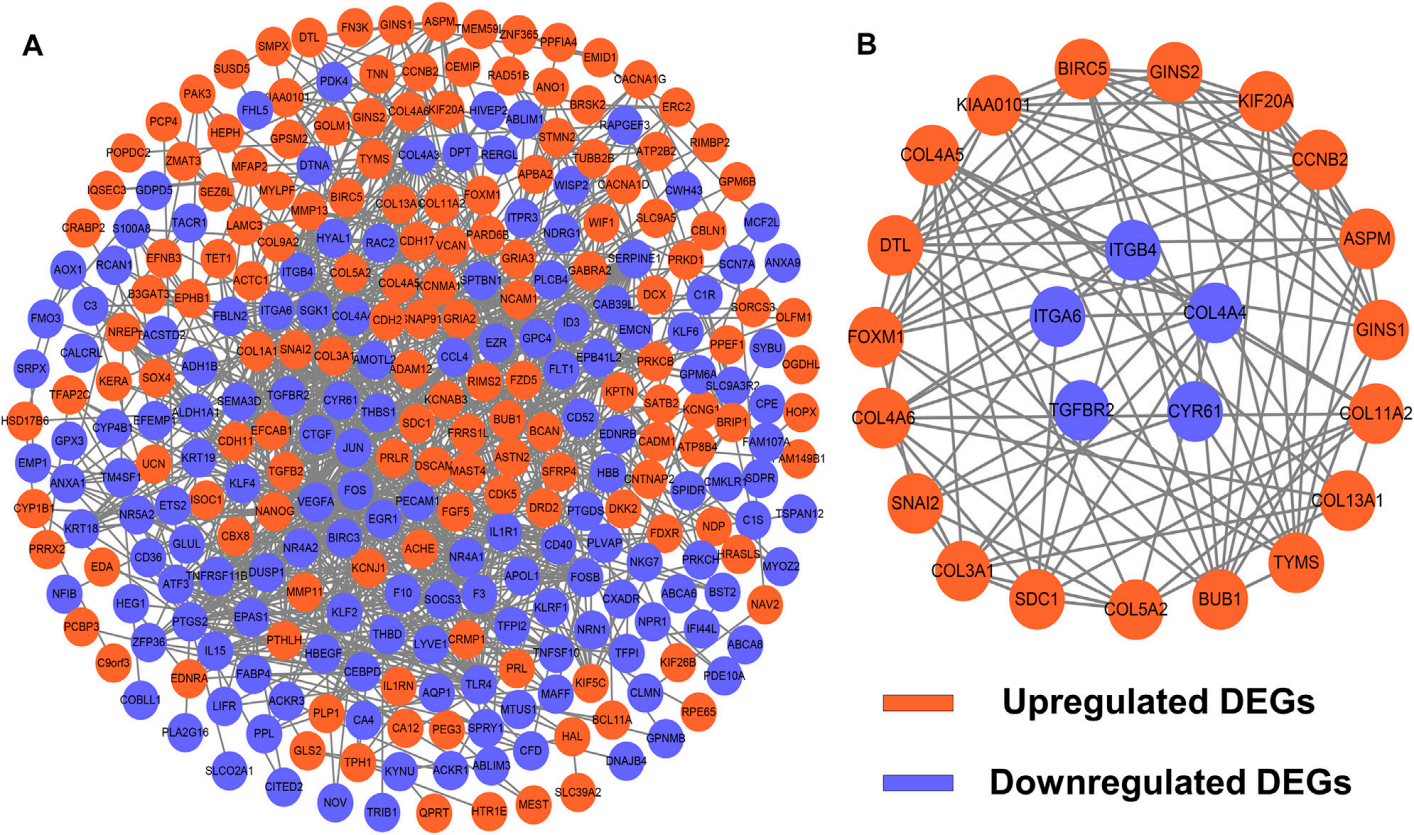
PPI network and module analysis. **(A)** 373 DEGs were visualized in the PPI network. **(B)** Module analysis using MCODE.

To identify potential interactions among 373 candidate DEGs, the PPI network was constructed using the STRING database and visualized by Cytoscape (Figure 3A). The network included 297 nodes (genes) and 1,111 edges (interactions). Furthermore, hub DEGs was validated by MCODE, and then we obtained 8 cluster modules. As indicated in Figure 3B, the highest-rated module included 24 nodes (genes) and 99 edges (interactions). The 24 hub DEGs included 19 upregulated and 5 downregulated genes.

### Enrichment Analysis of DEGs and Hub Genes

**FIGURE 4:**
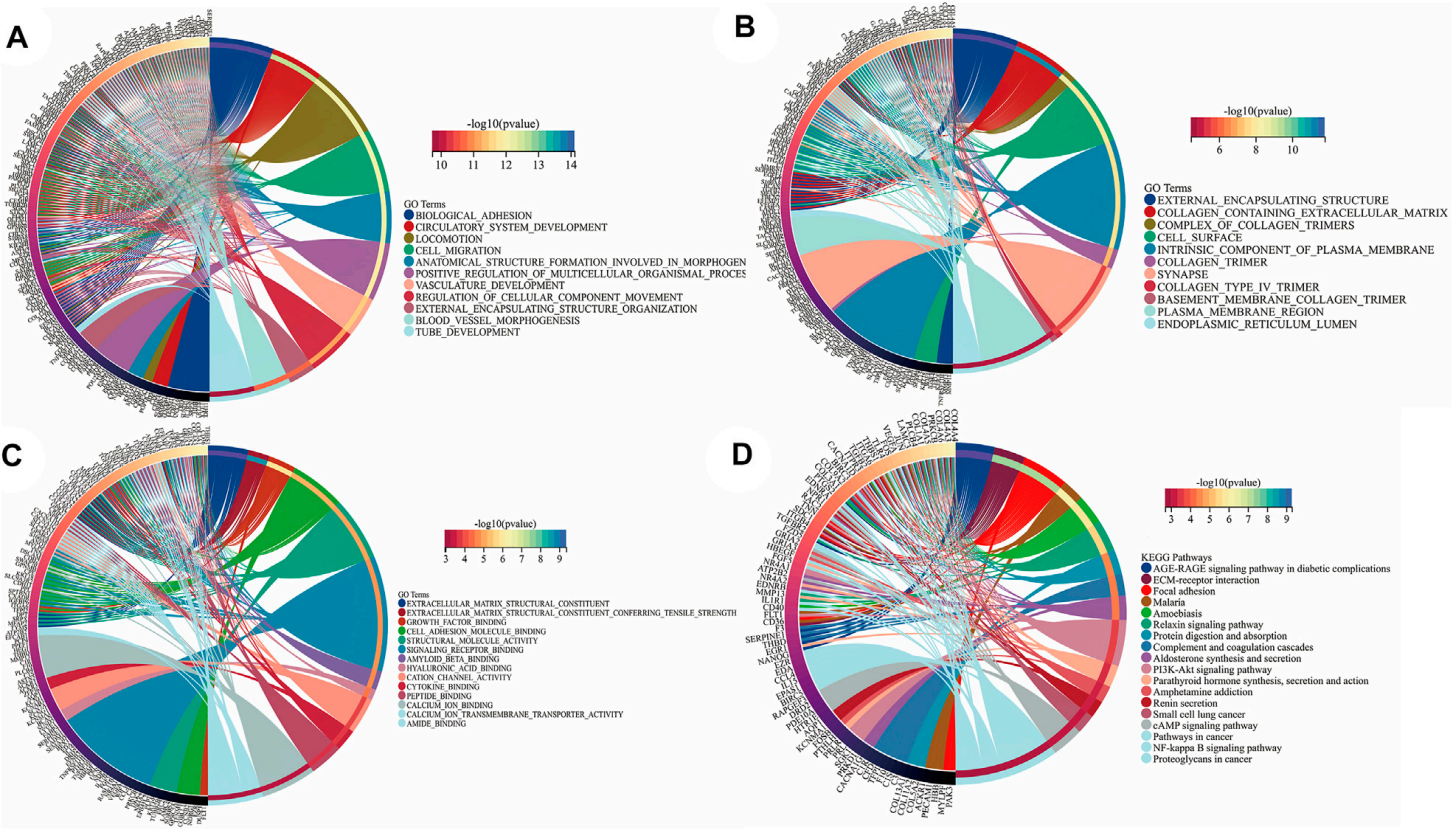
Function annotation and KEGG analysis of DEGs in UF. **(A)** BP-terms; **(B)** CC-terms; **(C)** MF-terms; **(D)** KEGG pathways.

To further reveal the vital function and biological pathways of DEGs and hub DEGs. We performed GO and KEGG analysis of 373 candidate DEGs and 24 hub DEGs. As illustrated in Figures 4A–D, for the BP-correlated category, DEGs were mainly enriched in biological adhesion, locomotion and cell migration; for CC-correlated category, DEGs were primarily involved in collagen containing extracellular matrix (ECM), an intrinsic component of the plasma membrane and cell surface; and for the MF-correlated category, DEGs were principally concerned with extracellular matrix structural consitituent, growth factor binding and signaling receptor binding. Furthermore, KEGG pathway results demonstrated that DEGs primarily participated in ECM-receptor interaction, focal adhesion, and the PI3K-AKT pathway.

**FIGURE 5:**
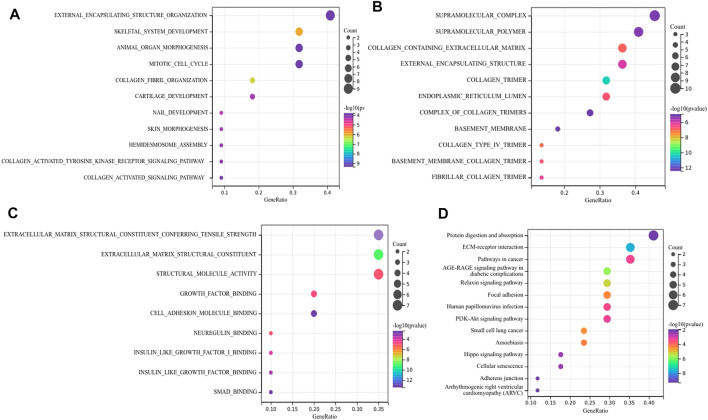
Function annotation and KEGG analysis of hub DEGs. **(A)** BP-terms **(B)** CC-terms; **(C)** MF-terms; **(D)** KEGG pathways.

As for hub DEGs, BP-enriched terms included external encapsulating structure organization and collagen fibril organization; CC-enriched terms were involved in supramolecule complexes and collagen trimers; MF-enriched terms referred to extracellular matrix structural consitituent and structural molecule activity. KEGG results contained ECM-receptor interactions and pathways in cancer (Figures 5A–D).

### Drug-Gene Networks

**FIGURE 6:**
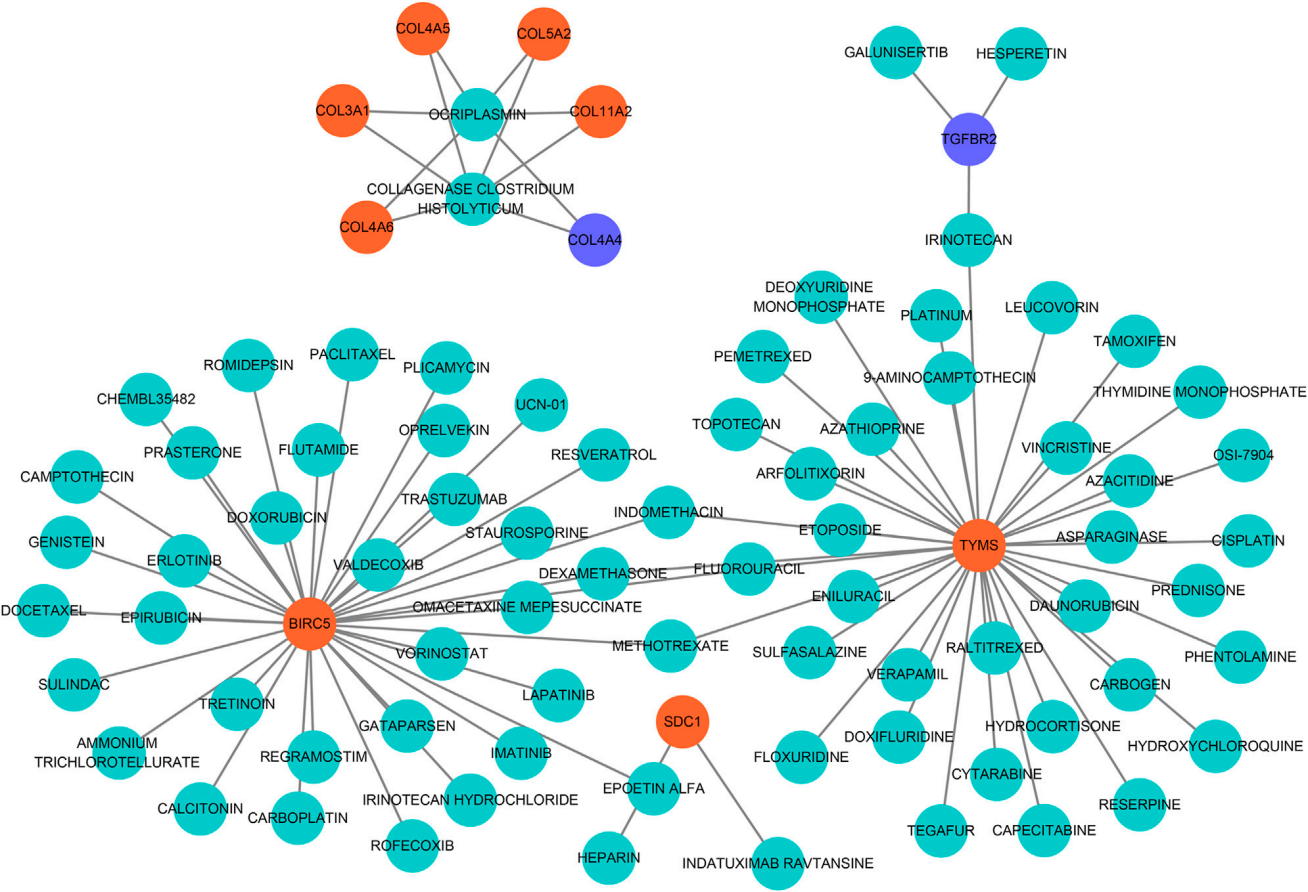
Drug-hub DEGs regulatory networks. Cyan circles, potential target drugs; Orange circles, upregulated DEGs; Purple circles, downregulated DEGs.

To seek effective target-drugs for hub DEGs, drug-gene interactions, including 75 potential drugs for UF, were obtained through the DGIdb database. For ease of reading, drug-gene networks were visualized by Cytoscape (Figure 6). However, the underlying mechanism between most potential drugs and hub DEGs remains unrevealed.

### Molecular Docking

**FIGURE 7:**
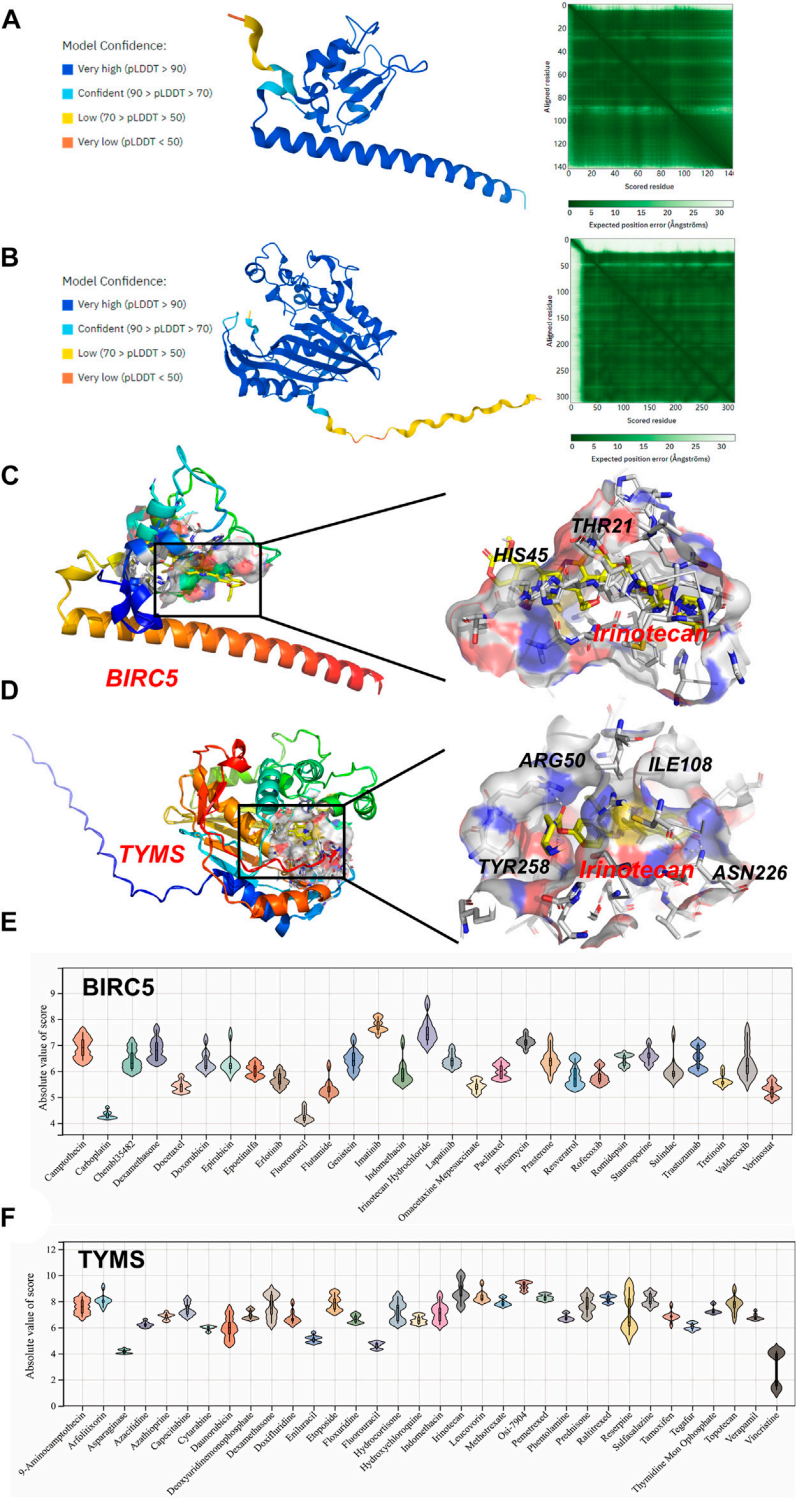
Homology Modeling and Molecular Docking. The crystal structure and evaluation of **(A)** BIRC5 and **(B)** TYMS. The best docking position between irinotecan and **(C)** BIRC5 or **(D)** TYMS was indicated. The absolute value of affinity between predicated small molecules and **(E)** BIRC5 or **(F)** TYMS was exhibited.

Molecular docking was applied to further screen potential drugs and elucidate possible molecular mechanisms. The crystal structure of human BIRC5 and TYMS was predicated by Alphafold v2.0 based on AI technology. As shown in Figures 7A,B, the stereochemical quality of the predicted structure was evaluated by the LDDT score. Autocdock Vina was used for drug-protein molecular docking to screen the optimal potential target drugs. The Affinity score can be used as a standard to judge the merits of docking. The high absolute value of the score indicated stronger binding between small molecules and proteins. The docking scores of potential drugs were presented in Figures 7C–F and suggested that irinotecan had the strongest binding affinity towards BIRC5 (7.0–8.6 kcal/mol, |interval score|) and TYMS (7.7–10.0 kcal/mol, |interval score|). The perfect conformation exhibited that irinotecan interacted with residues of BIRC5 (THR21 and HIS45) and TYMS (ARG50, ILE108, ASN226 and TYR258) through hydrogen bonds. After screening, irinotecan was found to be the optimal drug for intervention of both BIRC5 and TYMS and was used to treat UF patients in low doses.

### Validation of Hub DEGs by RT-PCR

**FIGURE 8:**
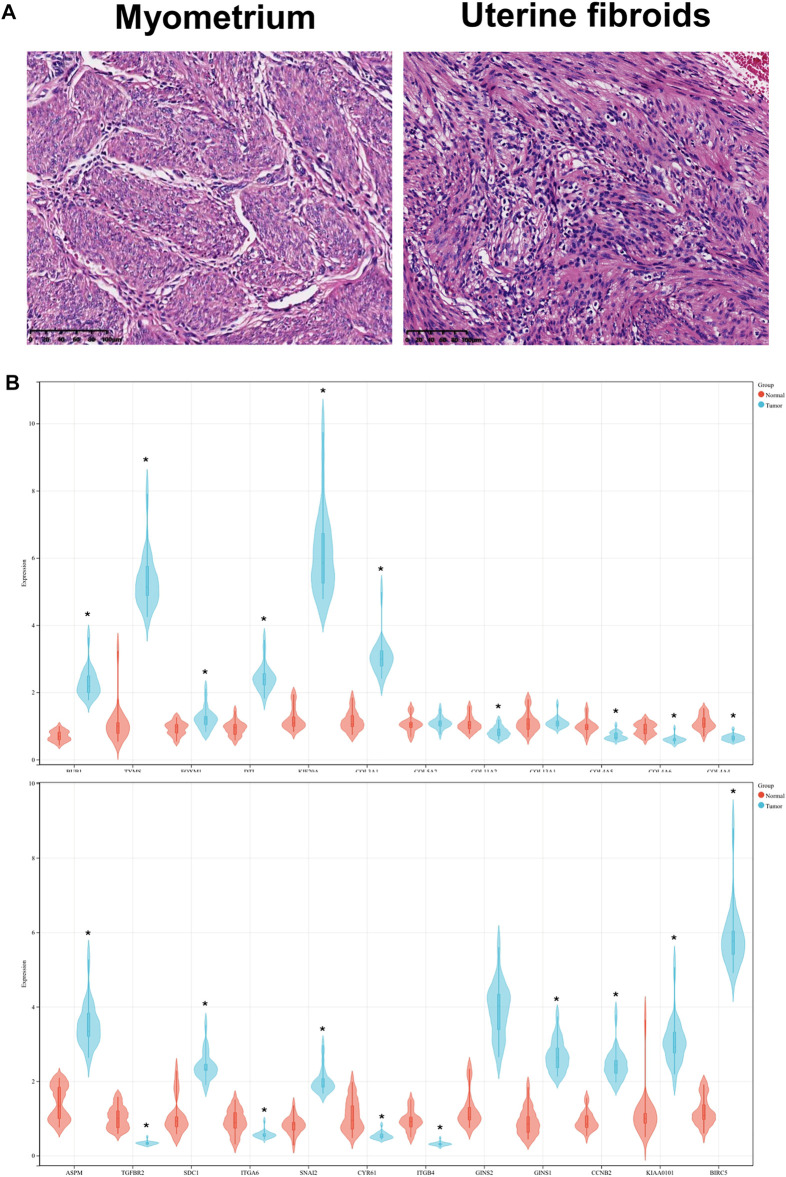
**(A)** HE staining of UF tissues and myometrium. **(B)** Validation of hub DEGs expression in UF by RT-PCR.

Based on UF and matched normal myometrial tissues in patients at our hospital, we validated the expression level of hub DEGs through RT-PCR (Figures 8A, B). Compared with normal myometrium, the expression levels of ASPM, SDC1, SNAI2, GINS1, CCNB2, KIAA0101, BIRC5, BUB1, TYMS, FOXM1, DTL, KIF20A, COL3A1 were increased, which was consistent with microarray analysis results. Moreover, the expression levels of TGFBR2, ITGA6, CYR61, ITGB4, COL11A2, COL4A5, COL4A6, and COL4A4 were decreased in comparison to normal myometrium. There were no significant differences in COL13A1 and COL5A2 expression.

### Prediction of Target Genes for DEMs and miRNA–Target Gene Regulatory Network Establishment

**FIGURE 9:**
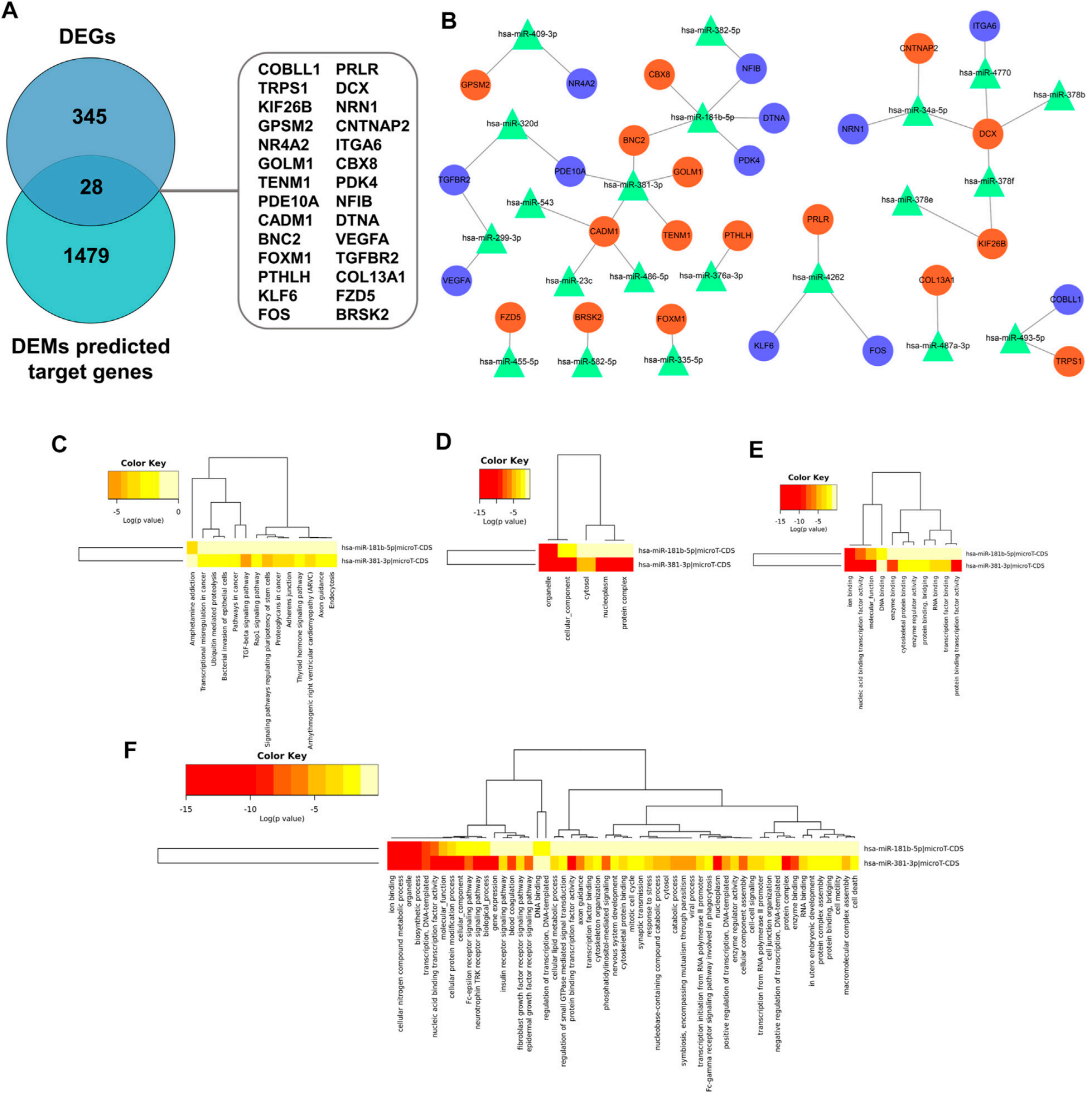
**(A)** Screen of DETGs of DEMs. The intersection of DEMs and predicted target genes by miRwalk3.0. **(B)** The miRNA-mRNA regulatory network. Triangles, DEMs; Orange circles, upregulated DETGs; Purple circles, downregulated DETGs. Function annotation and KEGG analysis of hub DEMs. **(C)** KEGG pathways; **(D)** CC-terms; **(E)** MF-terms; **(F)** BP-terms.

Recent research indicated that tumorigenesis and development were regulated by miRNAs through binding with pairing bases of 3′UTR, resulting in target mRNAs degradation. Therefore, predication of target mRNAs can contribute to revealing the function and internal mechanism of DEMs. MiRWalk 3.0 was used to predict the target genes of DEMs. A total of 1,507 genes were identified as the potential target genes of the DEMs. After intersecting with 373 candidate DEGs, 28 overlapping genes were identified as DETGs for further analysis (Figure 9A). The miRNA-target gene regulatory network was visualized using Cytoscape. As indicated in Figure 9B, triangle represents DEMs, orange ellipses represent upregulated DETGs, and purple ellipses represent downregulated DETGs. The regulatory network included 21 DEMs, 16 upregulated DETGs, and 12 downregulated DETGs. The DEMs were ranked by the number of regulated DETGs, and the top two were identified as hub DEMs. In vitro and vivo experiments validated the upregulated hsa-miR-181b-5p and hsa-miR-381-3p as well as five screened DETGs.

### Functional and Pathway Analysis of Hub DEMs

GO and KEGG pathway analysis were performed to elucidate the function of hub DEMs through miRPath v3.0. As illustrated in Figures 9C–F, these hub DEMs were enriched in ion binding, cellular nitrogen compound metabolic process, organelle, biosynthetic process and transcription, DNA-templated in the BP terms; Organelle and cellular component in CC terms; Ion binding, nucleic acid binding transcription factor activity and molecular function in MF terms. Furthermore, KEGG results indicated that hsa-miR-181b-5p and hsa-miR-381-3p involved in amphetamine addiction and TGF-β signaling pathway and proteoglycans in cancer respectively.

### Construction of the miRNA-TF-Target Gene Regulatory Network

**FIGURE 10:**
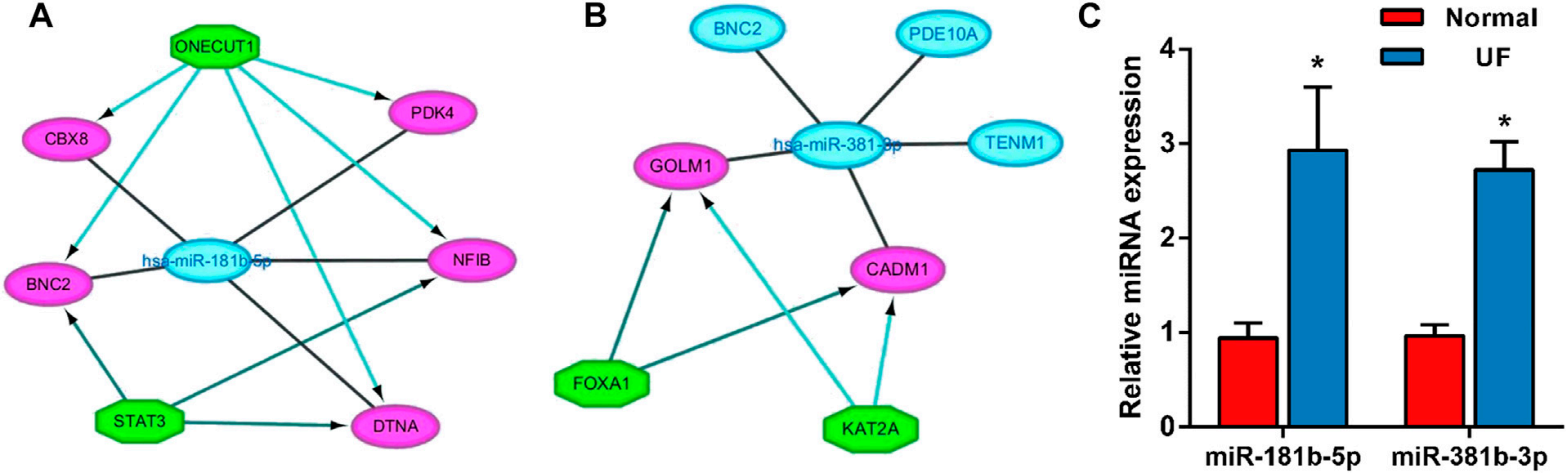
The miRNA-TF-mRNA regulatory networks of **(A)** hsa-miR-181-5p (2 TFs) and **(B)** hsa-miR-381-3p (2 TFs). **(C)** Expression of the miR-181b-5p, miR-381b-3p in UF tissues (*n* = 5) compared to normal myometrium tissues (*n* = 5), as assessed by reverse transcription–quantitative polymerase chain reaction.

The transcriptional regulatory relationships among these miRNAs, TFs, and target genes were identified through the iRegulon plug-in. Then, miRNA-TF-target gene networks were established through Cytoscape software. In the transcriptional regulatory network, hsa-miR-181b-5p consisted of 2 TFs (STAT3 and ONECUT1) and hsa-miR-381-3p consisted of 2 TFs (FOXA1 and KAT2A) (Figures 10A, B).

### Validation of Hub DEMs

In order to assess the expression of miR-181b-5p and miR-381b-3p in UF, RT-PCR was performed in UF tissues compared to normal myometrial tissues (*n* = 5). Consistent with our analysis of hub DEMs, we found that miR-181b-5p and miR-381b-3p were markedly upregulated in UF tissues compared to normal myometrium tissues (Figure 10C).

### The Effect of Hub DEMs on Cell Proliferation

**FIGURE 11:**
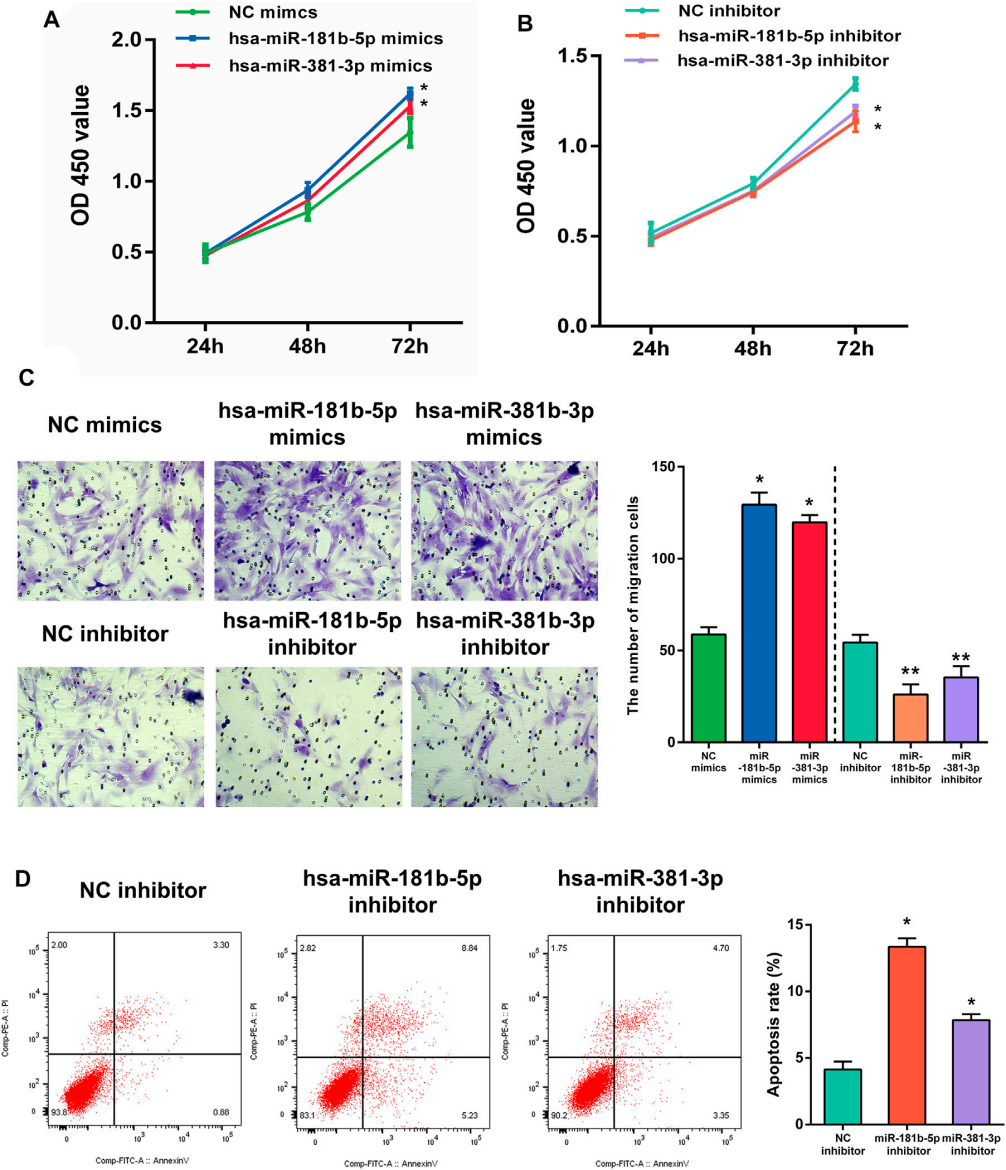
The proliferation of UF cells regulated by **(A)** overexpression or **(B)** slience of hub DEMs was detected by CCK-8 assay. **(C)** The migrational ability of UF cells regulated by overexpression or slience of hub DEMs was detected by Transwell. **(D)** The apoptosis rates of UF cells regulated by overexpression or slience of hub DEMs was detected by AV/PI assay.

In the functional verification section, the hub DEMs, mimics, and inhibitors were transfected into UF cells. The CCK-8 assay was used to examine the effects of hsa-miR-181b-5p and hsa-miR-381-3p on cell proliferation. As demonstrated in Figures 11A,B, overexpression of both hub DEMs enhanced the proliferation of UF cells compared with NC mimics, while hsa-miR-181b-5p and hsa-miR-381-3p inhibitors could reverse the above results.

### The Effect of Hub DEMs on Cell Migration

Although UF is a benign tumor with poor metastatic ability, it may locally migrate. Therefore, the evaluation of cell migration still reflects hub DEMs’ function. Many studies have also shown that UF cells have the metastatic ability. After transfection with hsa-miR-181b-5p and hsa-miR-381-3p mimics, the number of migrational cells was higher than NC mimic group. On the contrary, the metastatic ability of transfected cells with hsa-miR-181b-5p and hsa-miR-381-3p inhibitors was remarkably reduced, with fewer cells crossing through the membranes. Above, results suggested that there were positive correlations between the metastatic ability of UF cells and hsa-miR-181b-5p and hsa-miR-381-3p expression (Figure 11C)

### The Effect of Hub DEMs on Cell Apoptosis

Although surgical excision is the undisputed treatment for benign tumors, apoptosis of tumors induced by non-invasive procedures can effectively avoid operative trauma and tends to improve the outcome. MiRNA therapeutics provides a novel horizon for tumor treatment. To verify whether hub DEMs’ silence can induce UF cells’ apoptosis, we transfected NC, hsa-miR-181b-5p, and hsa-miR-381-3p inhibitors to UF cells. As indicated in Figure 11D. Downregulation of hsa-miR-181b-5p and hsa-miR-381-3 could significantly contribute to cell apoptosis through comparison of cell apoptosis rates in each group by flow cytometry.

## Discussion

As the most common benign tumor of the female reproductive system, the worldwide prevalence of UF has been increasing year by year (Pitter et al., 2013). Although UF is rarely malignant, it is often associated with menstrual changes, abdominal pain, abortions, and infertility (Giuliani et al., 2020). In addition, hysterectomy caused by UF seriously reduces the quality of life and places a heavy burden on basic social services such as health care (Cardozo et al., 2012). Unlike other types of cancer that have clear origins, there is a long-standing debate about whether the origin of UF is the immature smooth muscle cell derived from the uterine wall or the walls of uterine vessels (Akhter et al., 2021). Therefore, UF treatment cannot be applied to other tumor targets, indicating that screening of hub miRNAs and mRNAs associated with UF is urgently needed. Besides, the synergistic effects of TFs and miRNAs have become a new hot research topic for uncovering the etiology of diseases (Ciebiera et al., 2020). Unfortunately, to our knowledge, there is no correlated report establishing miRNA-TF-mRNA regulatory networks of UF. As pioneers, we first revealed internal interactions among miRNAs, TFs, and then mRNAs and revisited UF therapy from a novel perspective.

From the mRNA expression profile, 373 candidate DEGs were screened by taking the intersection of the two mRNA datasets. The sangerbox database was utilized to reveal the function and signaling pathway of DEGs. In the results of enrichment analysis, DEGs were closely associated with extracellular matrix structural constituents and biological adhesion, which was consistent with histopatological features of UF. Moreover, KEGG analysis also indicated that pathways enriched by DEGs are involved in ECM-receptor interaction, focal adhesion, and the PI3K-AKT pathway. In these DEGs, cysteine-rich angiogenic inducer 61 (CYR61) was widely reported to be correlated to UF (Sampath et al., 2001) (Arslan et al., 2005). Interesting, as an insulin-like growth factor (IGF)-binding protein, CYR61 degradation could promote IGF2 binding to IGF1R, leading to proliferation acceleration, ECM formation, and apoptosis inhibition (Sarkissyan et al., 2014).

To reduce the scope of DEGs, we established a PPI network and screened 24 hub DEGs, each containing 5 upregulated DEGs and 19 downregulated DEGs. Although most of these genes were subtypes of collagen, these data are also in line with the most striking pathological feature of UF (Bulun, 2013), excessive accumulation of ECM composed of collagen, resulting in irregular bleeding, pelvic pain, and compression. The ECM provides a microenvironment for cell adhesion, proliferation, and migration. Unfortunately, the internal interaction between ECM and UF cells remains unknown and is a hot issue to investigate deeply. Furthermore, as an essential transcriptional activator, aberrant SNAI2 can induce cell proliferation, invasion, and the epithelial-mesenchymal transition (EMT) process in a variety of malignancies (Casas et al., 2011). In gastric and lung cancers, abnormal expression of SNAI2 is associated with poor prognosis (Wang et al., 2021). Studies also indicated that the increased expression level of SDC1 could promote the growth, invasion and metastasis of tumor cells (Cao et al., 2021). FOXM1 plays a critical role in the regulation of cell proliferation, migration, and angiogenesis (Liu et al., 2021). It has been found that FOXM1 plays a vital role in mitosis, and its abnormal expression can cause spindle defects, which can delay mitosis and ultimately participate in the development of cervical (Li et al., 2021), liver (Nandi et al., 2021), lung (Liang et al., 2021) and other cancers. To validate the expression level of hub DEGs, we performed RT-PCR based on collected samples from our hospital. Most of the results on gene expression were in accordance with microarray results and provided corroborative evidence for our analysis.

Although limited drugs, such as GnRH (Dababou et al., 2021), mifepristone (Bi et al., 2021), are widely used in the treatment of UF, they lack a well-defined target and always bring patients side-effects (for example, recidivation after drug withdrawal). Therefore, target drugs for UF urgently need to be discovered to avoid physical injury caused by hysterectomy. Based on predicted hub DEGs, 75 target drugs approved by the FDA were discovered through the DGIdb database. As a natural polyphenol with anti-tumor property, resveratrol is a potent inhibitor of the progesterone X receptor (PXR) and may inhibit UF cell proliferation by decreasing estrogen and progesterone levels or antagonizing their receptors (Singh et al., 2011). In addition, ocriplasmin is applied to treat vitreomacular adhesions through the hydrolysis of collagen (Al-Nawaiseh et al., 2021). Considering the large number of target drugs for BIRC5 and TYMS, we utilized molecular docking to select the best candidates. Interestingly, irinotecan had the strongest binding to BIRC5 and TYMS through hydrogen bond formation. We hoped that the above results could give clinicians more novel therapeutic strategies.

Over the last few decades, miRNAs became one of the most discussed issues and was deemed as a biomarker to achieve early screening and clinical treatment. Especially in recent years, the advent of the first miRNA drug (miravirsen) rekindled the topic of morbigenous miRNA screening (Zhang et al., 2021). Certainly, miRNAs are involved in the regulation of multiple activities of UF. We identified DEMs based on the GSE159959 dataset and obtained 28 DEGTs through overlapping between target genes predicted by miRwalk3.0 and DEGs. Additionally, the miRNA-mRNA regulatory network was constructed and miR-181b-5p and miR-381-3p were identified as hub DEMs. The function analysis of hub DEMs indicated that they were enriched in ion binding, cellular nitrogen compound metabolic processes, organelles, biosynthetic processes, and transcription. Furthermore, the KEGG pathway analysis exhibited hsa-miR-181b-5p and hsa-miR-381-3p involved in amphetamine addiction and TGF-β signaling pathway and proteoglycans in cancer respectively.

Dysregulated miR-181b-5p was observed in various cancers and influenced the function of tumor cells. A previous study demonstrated that upregulated miR-181b-5p could promote cell proliferation, migration, and invasion by targeting PARK2 via the PTEN/PI3K/AKT pathway in cholangiocarcinoma (Jiang et al., 2021). Additionally, miR-181b-5p is also closely linked to drug resistance for gallbladder carcinoma (Wu et al., 2019). The potency of ginsenoside Rg3 could be abolished by miR-181b-5p through enhancing autophagy flux *via* the CREBRF/CREB3 pathway (Wu et al., 2019). Wu et al. also reported that miR-181b-5p was verified to be a biomarker for pituitary adenoma (Wu et al., 2017). Similarly, miR-181b-5p upregulation was involved in head and neck cancer pathogenesis (Nurul-Syakima et al., 2011). Nakajima G et al. found that miR-181b-5p was overexpressed in colon cancer tissues and promoted the occurrence and development of tumors by regulating cell signal transduction and cell cycle (Nakajima et al., 2006). Taken together, we have reasons to believe that miR-181b-5p plays a vital role in tumorigenesis and the progression of UF.

MiR-381-3p was reported as an oncogenic regulator involving in a wide range of cancers. Zhao et al. indicated that miR-381-3p overexpression could accelerate cell proliferation and colony formation, which further initiates renal tumor deterioration and poor prognosis (Zhao et al., 2020). A gynecologic cancer-associated study also elucidated that miR-381-3p had a positive correlation with uterine corpus endometrial carcinoma (Zheng et al., 2021). Furthermore, some studies showed that miR-381-3p was upregulated in glioma (Li et al., 2016), osteosarcoma (Li et al., 2016), synovial sarcoma (Hisaoka et al., 2011) and epithelioid sarcoma (Papp et al., 2014). Therefore, we hypothesized that miR-381-3p may become a clinical diagnostic and therapeutic target of UF. Definitely, due to spatial heterogeneity in tumor tissue, miR-381-3p was regarded as a tumor suppressor in gastric cancer (Gao et al., 2022), breast cancer (Yu et al., 2021) and bladder cancer (Li et al., 2019). We performed *in vitro* experiments to examine the function of miR-381-3p on UF, and the results verified our hypothesis.

To investigate the synergistic effect of TFs, miRNA-TF-mRNA regulatory networks of miR-181b-5p and miR-381-3p were established. miR-181b-5p and 2 TFs, STAT3 and ONECUT1, were verified to be coregulators of CBX8, PDK4, NFIB, BNC2, and DTNA. miR-381-3p and 2 TFs, FOXA1 and KAT2A, were verified to be coregulators of GOLM1, TENM1, PDE10A, CADM1, and BNC2. These two networks provided a novel horizon to explain synergistic interactions between miRNAs and TFs and potential combined treatments for UF.

RT-PCR was carried out to detect hub DEG expression in myometrium and uterine fibroids, RT-PCR was carried out. Compared with normal myometrium, the expression levels of ASPM, SDC1, SNAI2, GINS1, CCNB2, KIAA0101, BIRC5, BUB1, TYMS, FOXM1, DTL, KIF20A, COL3A1 were increased, which was consistent with microarray analysis results. Moreover, the expression levels of TGFBR2, ITGA6, CYR61, ITGB4, COL11A2, COL4A5, COL4A6, COL4A4 were decreased in comparison to normal myometrium. There were no significant differences in COL13A1 and COL5A2 expression.

Furthermore, in order to explore the effect of hub DEMs on UF cell proliferation, migration, and apoptosis, hsa-miR-181-5p and hsa-miR-381-3p were overexpressed or silenced and delivered into UF cells. CCK-8 demonstrated that hsa-miR-181-5p and hsa-miR-381-3p overexpression enhanced cell proliferation and migration in uterine fibroids cells compared with NC mimics, while silencing of hsa-miR-181-5p and hsa-miR-381-3p reversed the above results. Furthermore, we examined UF cells transfected with hub DEMs’ inhibitor. Downregulation of hsa-miR-181b-5p and hsa-miR-381-3p could significantly contribute to cell apoptosis in comparison of cell apoptosis rates of NC inhibitor by flow cytometry. Therefore, the function of hsa-miR-181b-5p and hsa-miR-381-3p deserves further study to elucidate the internal mechanism in UF.

Certainly, our study inevitably has some limitations. Although we performed *in vitro* experiments through overexpression and silence of hub DEMs, *in vivo* experiments could better simulate tumorigenesis and progression of UF to verify their function. Additionally, we did not assess the expression of target genes upon transfection. Therefore, our further study will conduct RT-PCR to evaluate the expression of target genes and establish an animal model to reveal the underlying mechanism of hub DEMs in UF. Screening chip data from the same platform can effectively avoid batch effects. In addition, miRNA is a type of noncoding RNA that includes lncRNAs, circRNAs etc. Whether other noncoding RNAs also play a vital role in UF is worth discussing. We hope to discover more noncoding RNAs as specific biomarkers to enhance the accuracy of early screening and improve patients’ outcomes.

## Conclusion

In conclusion, we identified some specific biomarkers (DEGs and DEMs) associated with the diagnosis and prognosis of UF based on bioinformatics analysis. Next, hub DEGs were confirmed by RT-PCR of clinical UF samples. Furthermore, we first established miRNA-TF-mRNA regulatory networks of UF and screened hsa-hsa-miR-181-5p and hsa-miR-381-3p as hub DEMs that are associated with regulation of cell proliferation, migration, and apoptosis in UF through *in vitro* validation. Our study provided several novel biomarkers to reveal possible mechanisms and was believed to be helpful for the diagnostic and therapeutic strategy of UF.

## Data Availability

The datasets presented in this study can be found in online repositories. The names of the repository/repositories and accession number(s) can be found below: https://www.ncbi.nlm.nih.gov/geo/, GSE593; https://www.ncbi.nlm.nih.gov/geo/, GSE18096; https://www.ncbi.nlm.nih.gov/geo/, GSE159959.
